# The LAUsanne *STAPHylococcus aureus* ENdocarditis (LAUSTAPHEN) score: A prediction score to estimate initial risk for infective endocarditis in patients with *S. aureus* bacteremia

**DOI:** 10.3389/fcvm.2022.961579

**Published:** 2022-12-09

**Authors:** Matthaios Papadimitriou-Olivgeris, Pierre Monney, Linda Mueller, Laurence Senn, Benoit Guery

**Affiliations:** ^1^Infectious Diseases Service, Lausanne University Hospital and University of Lausanne, Lausanne, Switzerland; ^2^Infection Prevention and Control Unit, Lausanne University Hospital and University of Lausanne, Lausanne, Switzerland; ^3^Department of Cardiology, Lausanne University Hospital and University of Lausanne, Lausanne, Switzerland; ^4^Institute of Microbiology, Lausanne University Hospital and University of Lausanne, Lausanne, Switzerland

**Keywords:** *Staphylococcus aureus* bacteraemia, infective endocarditis, transoesophageal echocardiography (TOE), bloodstream infection, risk stratification

## Abstract

**Introduction:**

Infective endocarditis (IE) is a common complication of *Staphylococcus aureus* bacteremia (SAB). The study aimed to develop and validate a prediction score to determine IE risk among SAB.

**Methods:**

This retrospective study included adults with SAB (2015–2021) and divided them into derivation and validation cohorts. Using the modified 2015 European Society of Cardiology modified Duke Criteria for definite IE, the LAUSTAPHEN score was compared to previous scores.

**Results:**

Among 821 SAB episodes, 419 and 402 were divided into derivation and validation cohorts, respectively. Transthoracic and transoesophageal echocardiography (TOE) were performed in 77.5 and 42.1% of episodes, respectively. Definite IE was diagnosed in 118 episodes (14.4%). Derivation cohort established that cardiac predisposing factors, such as cardiac implantable electronic devices, prolonged bacteremia ≥48 h, and vascular phenomena were independently associated with IE. In addition to those parameters, native bone and joint infections were used to constitute the LAUSTAPHEN score. LAUSTAPHEN and VIRSTA scores misclassified <4% of IE cases as low risk. Misclassification using POSITIVE and PREDICT scores was >10%. The number of TOEs required to safely exclude IE were 66.9 and 51.6% with VIRSTA and LAUSTAPHEN, respectively.

**Discussion:**

LAUSTAPHEN and VIRSTA scores exhibited the lowest misclassification rate of IE cases to the low-risk group. However, the number of patients requiring TOE was higher for VIRSTA than for LAUSTAPHEN.

## Introduction

*Staphylococcus aureus* is one of the leading causes of bacteremia in both community and nosocomial-acquired infections. *S. aureus* bacteremia (SAB) is associated with increased mortality which is influenced by the presence of metastatic foci, such as infective endocarditis (IE) (1–6). IE is estimated to complicate 10–20% of SAB (7–11).

According to the guidelines of IE management (12, 13), echocardiography should be performed in all episodes of SAB to exclude IE. Transoesophageal echocardiography (TOE) is preferred to transthoracic (TTE), due to a higher sensitivity. Nevertheless, being an invasive procedure, TOE cannot be performed in all patients (12, 14). Indeed, the development of scores to identify patients in low- and high-risk groups for IE is warranted to avoid unnecessary echocardiograms (15–17). Among several clinical prediction rules proposed so far, VIRSTA, Predicting Risk of Endocarditis Using a Clinical Tool (PREDICT), and POSITIVE scores were recently developed, with VIRSTA showing the best diagnostic accuracy (8, 10, 15, 18).

VIRSTA score included 10 parameters (cerebral or peripheral emboli, meningitis, vertebral osteomyelitis, permanent intracardiac device or previous IE, pre-existing native valve disease, intravenous drug use, prolonged bacteremia for 48 h, community or non-nosocomial healthcare-associated bacteremia, severe sepsis or shock, and C-reactive protein >190 mg/L) with each parameter ranging from 1 to 5 points (8). PREDICT score included three parameters (implantable cardioverter defibrillator or permanent pacemaker, community or healthcare acquisition, and prolonged bacteremia for 72 h) with each parameter ranging from 1 to 3 points (19). Finally, the POSITIVE score included four parameters (time to blood culture positivity, intravenous drug use, cerebral or peripheral emboli, and predisposing heart disease) with each parameter ranging from 2 to 6 points (10). The main drawbacks of the aforementioned scores were the need for complex calculations (multiple parameters with different ranges of each parameter), the fact that all scores contain several variables included in the Duke criteria, and that all scores were calculated and validated in populations that not all patients benefited from echocardiograms (8–11, 19, 20).

This study aimed to develop and validate a new simple prediction rule to stratify the risk of IE within 72 h from SAB onset and to compare it with other existing scores (PREDICT, POSITIVE, and VIRSTA).

## Materials and methods

### Study design

This retrospective study was conducted at the Lausanne University Hospital, Lausanne, Switzerland, with an 1100-bed primary and tertiary care hospital, during a 7-year period (2015–2021). The study was approved by the ethics committee of the Canton of Vaud (CER-VD 2021-02516) that waived the need for informed consent.

### Patients

Inclusion criteria were adult patients (≥18 years old) and the presence of at least one positive blood culture for *S. aureus* (extracted from the database of the microbiology laboratory). Exclusion criteria were patients' written refusal of the use of their data, incomplete medical files, and death within 48 h from bacteremia onset.

Data regarding demographics (age, sex), comorbidities, laboratory results (white blood cells, C-reactive protein), presence of sepsis or septic shock, foci of infection, individual components of 2015 European Society of Cardiology (ESC) modified Duke Criteria (12), cardiac imaging results, cardiac surgery or ablation of cardiac implantable electronic devices (CIEDs), autopsy results, and persistent bacteremia were retrieved from patients' electronic health records. Study data were collected and managed using REDCap by an infectious diseases specialist. REDCap electronic data capture tools are hosted at Lausanne University Hospital. Research Electronic Data Capture (REDCap) is a secure, web-based software platform designed to support data capture for research studies (18, 21).

### Management of SAB

According to the internal guidelines, an infectious diseases consultation was performed on a mandatory basis within the same day of blood culture positivity for *S. aureus*. According to the published evidence (12, 14, 17), our internal policy recommended TTE and TOE in patients with community-acquired bacteremia (Group 1). TTE and TOE were also suggested in the case of nosocomial bacteremia with risk factors for IE such as prior IE, presence of CIED or prosthetic valve, persistent BSI for 72 h, or embolic event (Group 2). For patients with nosocomial not catheter-related bacteremia without the aforementioned risk factors, only TTE was proposed (Group 3). Finally for nosocomial catheter-related bacteremia without risk factors, no further investigation was warranted (Group 4). Follow-up blood cultures at 48 h intervals were recommended until negativization.

### Definitions

The date of collection of the first positive blood culture was defined as infection onset. A new episode was included if more than 30 days had elapsed since the first negative blood culture of the initial episode. Bacteremia was characterized as a community, healthcare, or nosocomial according to Friedman et al. (22) Infection was categorized as sepsis or septic shock according to the definition proposed by the Sepsis-3 International Consensus (23). Definite IE was defined according to the 2015 ESC-modified Duke Criteria (12). Cardiac predisposing factors for IE were defined as cardiac conditions at high or moderate risk for IE (24). Vascular phenomena were defined as arterial, septic lung emboli, renal or splenic emboli, mycotic aneurysm, intracranial ischemia or bleeding, conjunctival bleeding, Janeway lesions, or nail bed bleeding.

### LAUsanne *STAPHylococcus aureus* ENdocarditis (LAUSTAPHEN) score

The population was divided into a derivation and a validation cohort according to the date of bacteremia onset (derivation cohort: first 6 months of each year; validation cohort: last 6 months of each year). Patients with definite IE were compared to those without (possible IE or rejected IE). Four variables (cardiac predisposing factors, CIED, prolonged bacteremia ≥48 h, vascular phenomena) were preselected for the model according to the clinical practice since their presence is highly associated with IE, leads physicians to suspect IE, and usually triggers further cardiac imaging investigations (15, 17). The primary aim was to reach < 4% of misclassified IE in the low-risk group in conjunction with minimizing the number of cardiac imaging studies needed to be performed. In case the four preselected variables did not suffice to attain the aforementioned threshold, a fifth variable would be selected from other variables known to be associated with IE, namely immunologic phenomena, native bone and joint infections (septic arthritis and vertebral and non-vertebral osteomyelitis) community and non-nosocomial healthcare-associated SAB, time to blood culture positivity < 9 h, meningitis, or septic shock (15). The associations between such variables and IE were measured by univariate analysis. For the identification of the best-performing model, multiple multivariable analyses were performed by including the four preselected variables and each of the aforementioned variables. The best-performing model from the derivation cohort was chosen. LAUSTAPHEN score's diagnostic accuracy was then tested in a separate validation cohort.

### Analysis

We used the predefined cut-offs of the evaluated scores (VIRSTA, POSITIVE, and day 5 PREDICT) for the identification of patients at low or high risk for IE (8, 10, 19). Episodes with VIRSTA score ≥3 (8), POSITIVE score ≥5 (10), and PREDICT score (on day 5) ≥2 (19) were considered at high risk for IE.

The POSITIVE score was not calculated in patients for whom time to positivity of blood cultures was missing (bacteremia onset in other hospitals) or unreliable (polymicrobial bacteremia, blood cultures collected while patient was on antimicrobial treatment). CRP missing values were imputed with the median value.

The primary endpoint was the diagnostic accuracy of LAUSTAPHEN and the aforementioned scores for the diagnosis of IE. Two analyses were performed; for the first one, the reference standard was definite IE according to the 2015 ESC-modified Duke Criteria, while, for the second, the reference standard was the presence of cardiac lesions detected with imaging and pathological examination according to the 2015 ESC-modified Duke Criteria (12). Sensitivity, specificity, positive and negative predictive values (PPV, NPV), and positive and negative likelihood ratios (PLR, NLR), as well as accuracy, were calculated. The number of patients with IE misclassified into low risk was calculated for each score, as well as the number of TOE indications resulting from the high-risk stratification by each score. Receiver operating curves were also generated.

SPSS version 26.0 (SPSS, Chicago, IL, USA) software was used for data analysis. Categorical variables were analyzed using the *chi*-square or Fisher exact test and continuous variables with Mann–Whitney *U-*test. Multivariable logistic regression analyses were performed in the derivation cohort by using two dependent variables; definite IE according to the 2015 ESC-modified Duke Criteria and presence of cardiac lesion according to imaging and pathological 2015 ESC-modified Duke Criteria. Variables that did not contribute to multicollinearity were used in multivariable analysis. Odds ratios (ORs) and 95% confidence intervals (CIs) were calculated to evaluate the strength of any association. All statistical tests were two-tailed, and *P* < 0.05 was considered statistically significant.

## Results

### Study population

Among the 1,060 episodes of SAB, 821 episodes in 762 patients were included in this study (Supplementary Figure 1). The derivation and validation cohorts comprised 419 and 402 patients, respectively. Supplementary Table 1 shows the baseline characteristics of derivation and validation cohorts.

In total, TTE was performed in 636 episodes (77.5%), while TOE was performed in 346 (42.1%). The timing from bacteremia onset to the first echocardiogram (either TTE or TOE) was 2 days (Q1–Q3: 0–4 days). Due to high clinical suspicion of IE, among 606 episodes with negative initial TTE, 79 had a second TTE performed leading to the establishment of the 2015 ESC-modified Duke imaging criterion in four episodes. Furthermore, among 290 episodes with negative initial TEE or TOE, a second TOE was performed due to high clinical suspicion of IE in 20 episodes, five of which fulfilled the 2015 ESC-modified Duke imaging criterion. Among the 606 episodes at high risk according to internal policy for IE (Groups 1 and 2; Supplementary Table 2), TOE was performed in 307 (50.7%) episodes. Although indicated, TOE was not performed in 299 episodes because of the following reasons: a combination of age and comorbidities (100 episodes; 33.4%), contraindication (esophageal varices and severe thrombocytopenia) or non-feasibility (severe obesity, inability to pass the endoscope, death before TOE) (44; 14.7%), and positivity of TTE (10; 3.3%); for the remaining 145 episodes (48.5%), the risk was deemed low by the treating physician or infectious diseases consultant; thus, no further testing was pursued. Other imaging modalities (18-FDG PET-CT, cardiac CT) were performed in 104 episodes (12.7%). In total, at least one cardiac imaging study was performed in 700 episodes (85.3%). According to internal guidelines, at least one cardiac imaging study was performed in 551 episodes (90.9%) among 606 categorized at high risk (Groups 1 and 2) and in 149 (69.3%) among 215 episodes categorized at low risk (Groups 3 and 4) (Supplementary Table 2).

### IE classification

Definite IE according to the clinical and pathological Duke criteria was diagnosed in 118 episodes (14.4%) (Supplementary Table 3). Surgery was performed in 36 patients (27 with pathological criterion), autopsy in 14 patients (3 with pathological criterion), and CIED removal in 29 patients among 88 with CIED (21 with pathological criterion). In 102 patients with cardiac lesions (imaging and pathological 2015 ESC-modified Duke Criteria), IE involved native valves in 69 episodes (67.6%), prosthetic valves in 25 episodes (24.5%), and CIED in 14 episodes (20.6%). Valvular lesions were detected by imaging studies, cardiac surgery, or autopsy in 84 patients (76 with valvular vegetations, 16 abscesses, 10 perforations, two intracardiac fistulas, and 16 abnormal activities in 18-FDG PET-CT). CIED lesions were detected with imaging studies and CIED removal in 21 patients.

### Predictors of IE

Table 1 shows the univariate and multivariable analyses of IE predictors in the derivation cohort with definite IE according to the 2015 ESC-modified Duke Criteria as the reference standard. In the univariate analysis, among other variables, patients with IE were more likely to have cardiac predisposing factors (*P* < 0.001), CIED (*P* < 0.001), prolonged bacteremia ≥48 h (*P* < 0.001), vascular phenomena (*P* < 0.001), and native bone and joint infections (septic arthritis and vertebral and non-vertebral osteomyelitis) (*P* 0.004). Multivariable analysis identified cardiac predisposing factors (*P* < 0.001; OR 6.4, CI 2.8–14.6), CIED (*P* < 0.001; OR 8.2, CI 3.2–20.9), prolonged bacteremia ≥48 h (*P* 0.035; OR 1.3, CI 1.0–1.7), and vascular phenomena (*P* < 0.001; OR 15.7, CI 7.2–34.4) as independent predictors of IE among patients with SAB.

**Table 1:** Predictors of definite infective endocarditis (according to the 2015 ESC-modified Duke Criteria) in patients with *S. aureus* bacteremia in the derivation cohort.

	**Univariate analysis**					**Multivariable analysis**	
	**Without IE (*****n** **=*** **362)**		**IE (*****n** **=*** **57)**		***P***	***P***	**OR (95% CI)**
**Demographics**							
Male sex	261	72.1%	46	80.7%	0.173		
Age (years)	67	55–79	60	48–70	0.003		
**Co-morbidities**							
Congestive heart failure	29	8.0%	8	14.0%	0.136		
Chronic obstructive pulmonary disease	40	11.0%	9	15.8%	0.301		
Cirrhosis	34	9.4%	2	3.5%	0.141		
Diabetes mellitus	108	29.8%	10	17.5%	0.055		
Chronic kidney disease (moderate or severe)	83	22.9%	12	21.1%	0.753		
Malignancy (solid organ or haematologic)	74	20.4%	2	3.5%	0.002		
Obesity	86	23.8%	14	24.6%	0.869		
Immunosuppression	75	20.7%	1	1.8%	< 0.001		
Charlson Comorbidity Index	5	3–7	3	0–7	0.001		
**Setting of infection onset**							
Community	140	38.7%	38	66.7%	< 0.001^a^		
Non-nosocomial healthcare-associated	85	23.5%	11	19.3%			
Nosocomial	137	37.8%	8	14.0%			
Cardiac predisposing factors	30	8.3%	37	64.9%	< 0.001	< 0.001	6.4 (2.8–14.6)
IV drug use	24	6.6%	12	21.1%	< 0.001		
Prior endocarditis	5	1.4%	2	3.5%	0.244		
Native valve disease	1	0.3%	3	5.3%	0.009		
Prosthetic valve	8	2.2%	13	22.8%	< 0.001		
Cardiac implantable electronic devices	28	7.7%	19	33.3%	< 0.001	< 0.001	8.2 (3.2–20.9)
Pacemaker	16	4.4%	10	17.5%			
Defibrillator	7	1.9%	4	7.0%			
Other	5	1.4%	5	8.8%			
**Presence of prosthetic material (other than cardiac valve)**							
Endovascular (non-cardiac) prosthetic material	20	5.5%	6	10.5%	0.147		
Bone or joint prosthetic material	83	22.9%	11	19.3%	0.541		
**Microbiological data**							
Two or more blood cultures positive	275	76.0%	57	100%	< 0.001		
Polymicrobial bacteraemia	35	9.7%	5	8.8%	1.000		
Methicillin-resistance	34	9.4%	0	0.0%	0.008		
Time to blood culture positivity (h) (among 396 patients)	13	10–17	11	8–15	0.004		
Time to blood culture positivity < 9 h	50	14.3%	22	46.8%	< 0.001^b^		
Time to blood culture positivity 9–11 h	80	22.9%	12	25.5%			
Time to blood culture positivity 11–13 h	57	16.3%	4	8.5%			
Duration of bacteraemia (h)	0	0–40	64	26–110	< 0.001		
Prolonged bacteraemia ≥48 h	76	21.0%	32	56.1%	< 0.001	0.035	1.3 (1.0–1.7)
Prolonged bacteraemia ≥72 h	46	12.7%	23	40.4%	< 0.001		
Imaging criterion	1	0.1%	88	74.6%	< 0.001		
TTE performed	270	74.6%	47	82.5%			
TOE performed	127	35.1%	42	73.7%			
18-FDG PET-CT or cardiac CT performed	42	11.6%	16	28.1%			
Any cardiac imaging performed	297	82.0%	55	96.5%			
**Infection data**							
Duration of systemic symptoms (days)	1	1–2	2	1–3	< 0.001		
Fever	295	81.5%	50	87.7%	0.252		
Heart murmur	104	28.7%	33	57.9%	< 0.001		
New heart murmur	61	16.9%	25	43.9%	< 0.001		
Vascular phenomena	29	8.0%	39	68.4%	< 0.001	< 0.001	15.7 (7.2–34.4)
**Location**							
Limbs	5	1.4%	10	17.5%	< 0.001		
Trunk	22	6.1%	27	47.4%	< 0.001		
Cerebral	7	1.9%	19	33.3%	< 0.001		
**Type**							
Ischemic stroke	6	1.7%	16	28.1%	< 0.001		
Hemorrhagic stroke	1	0.3%	3	5.3%	0.009		
Cerebral mycotic aneurysm	0	0.0%	0	0.0%	-		
Janeway lesions	2	0.6%	7	12.3%	< 0.001		
Nail bed hemorrhage	0	0.0%	2	3.5%	0.018		
Conjunctival bleeding	0	0.0%	0	0.0%	-		
Septic lung emboli	11	3.0%	14	24.6%	< 0.001		
Renal emboli	4	1.1%	10	17.5%	< 0.001		
Splenic emboli	6	1.7%	11	19.3%	< 0.001		
Non-cerebral mycotic aneurysm	8	2.2%	4	7.0%	0.066		
Arterial emboli	1	0.3%	2	3.5%	0.050		
**Other foci of infection**							
Meningitis	0	0.0%	2	3.5%	0.018		
Bone and joint infection (excluding chronic osteomyelitis)	88	24.3%	19	33.3%	0.146		
Native bone and joint infection (excluding chronic osteomyelitis)	62	17.1%	19	33.3%	0.004	0.160	1.9 (0.8–4.5)
Native septic arthritis	32	8.8%	12	21.1%	0.005		
Vertebral osteomyelitis	31	8.6%	8	14.0%	0.186		
Acute non-vertebral osteomyelitis	6	1.7%	2	3.5%	0.298		
Prosthetic bone and joint infection	30	8.3%	1	1.8%	0.101		
Prosthetic joint infection	25	6.9%	0	0.0%	0.035		
Osteosynthesis or spondylodesis infection	5	1.4%	1	1.8%	0.586		
Immunologic phenomena	2	0.6%	5	8.8%	0.001		
Sepsis	147	40.6%	30	52.6%	0.088		
Septic shock	42	11.6%	20	35.1%	< 0.001		
**Laboratory data**							
White blood cells ( × 10^9^/l)	12.2	7.9–17.1	13.1	10.5–16.5	0.297		
CRP (mg/l) (among 392 patients)	195	94–288	248	149–320	0.004		
CRP≥190mg/l	173	51.2%	34	63.0%	0.107		
**Management**							
Infectious diseases consultation	341	94.2%	56	98.2%	0.203		
Cardiac surgery	0	0.0%	20	35.1%	< 0.001		
CIED removal (among 47 patients with CIED)	4	14.3%	9	4.4%	0.020		
Autopsy (within 30 days)	5	1.4%	3	5.3%	0.081		

Data are depicted as number and percentage or median and Q1–3.

^a^Comparison of community-acquired against both non-nosocomial healthcare-associated and nosocomial.

^b^Comparison of time to blood culture positivity < 9 h against ≥9.

18-FDG PET-CT, 18-fluorodeoxyglucose positron emission tomography-computed tomography; CIED, cardiac implantable electronic devices; CRP, C-reactive protein; ESC, European Society of Cardiology; IE, infective endocarditis; TTE, transthoracic echocardiography; TOE, transoesophageal echocardiography.

Supplementary Table 4 shows the univariate and multivariable analyses of predictors of cardiac lesions according to imaging and pathological 2015 ESC-modified Duke Criteria in the derivation cohort. In the univariate analysis, among other variables, patients with cardiac lesions were more likely to have cardiac predisposing factors (*P* < 0.001), CIED (*P* < 0.001), prolonged bacteremia ≥48 h (*P* < 0.001), vascular phenomena (*P* < 0.001), and native bone and joint infections (*P* 0.002). Multivariable analysis revealed cardiac predisposing factors (*P* 0.002; OR 3.5, CI 1.6–7.7), CIED (*P* < 0.001; OR 5.8, CI 2.5–13.7), prolonged bacteremia ≥48 h (*P* 0.029; OR 1.3, CI 1.0–1.7), and vascular phenomena (*P* < 0.001; OR 8.9, CI 4.2–18.7) as independent predictors of cardiac lesion among patients with SAB.

### Development of LAUSTAPHEN score

Based on the derivation cohort, we developed a new prediction score aiming to reduce the false-negative rate (misclassified IE in the low-risk group) to < 4%, while minimizing the number of cardiac imaging procedures. A model including the four preselected variables (cardiac predisposing factors, CIED, prolonged bacteremia ≥48 h, and vascular phenomena) did not allow for reaching the false-negative cut-off. Thus, to reach the aforementioned threshold, additional models were tested by adding one of the several clinically relevant parameters with a high association with IE (immunologic phenomena, native bone and joint infections, community and non-nosocomial healthcare-associated SAB, time to blood culture positivity < 9 h, and meningitis or septic shock) to the existing four-item model. Among the aforementioned parameters, only the addition of community and non-nosocomial healthcare-associated SAB or native bone and joint infections to the four preselected variables improved the score's performance by achieving a false-negative rate of < 4%. Among the two variables, the presence of native bone and joint infections (septic arthritis, vertebral and non-vertebral osteomyelitis) was then added as a fifth item despite the absence of an independent association with IE in the multivariable model. The decision was based on the fact that the addition of community and non-nosocomial healthcare-associated SAB would have required more cardiac imaging investigations to achieve the same result as native bone and joint infections. The presence of any of the five parameters (cardiac predisposing factors, CIED, prolonged bacteremia ≥48 h, vascular phenomena, and native bone and joint infections) included in the LAUSTAPHEN score at 96 h from the onset of bacteremia classified patients as high-risk; one point was given for the presence of each variable (Table 2).

**Table 2:** Overview of LAUSTAPHEN score (cut-off ≥1 point).

**Items**	**Point assigned**
Cardiac predisposing factors (24)	1
Cardiac implantable electronic device	1
Prolonged bactereamia (≥48 h)	1
Vascular phenomena (12)	1
Native bone and joint infections	1

### Diagnostic accuracy of LAUSTAPHEN, PREDICT, VIRSTA, and POSITIVE scores

Table 3 shows the diagnostic accuracies of LAUSTAPHEN (in both, derivation and validation cohorts), PREDICT, VIRSTA, and POSITIVE scores in predicting definite IE. LAUSTAPHEN and VIRSTA scores had a misclassification rate of IE in the low-risk group of < 4% and an NPV of >98%. The NPV of PREDICT and POSITIVE scores was >95%; however, 13.6% and 23.5% of episodes of IE were misclassified in the low-risk group by PREDICT and POSITIVE scores, respectively. If the prediction scores had been applied to the whole study population, VIRSTA would have required more cardiac imaging investigations (TOE in 66.9% episodes with SAB) compared to LAUSTAPHEN (TOE in 51.6%) to achieve the same result (an increase of 29.7% of TOE needed). Table 4 shows the diagnostic accuracies of the aforementioned scores in predicting cardiac lesions. Results were similar, with LAUSTAPHEN and VIRSTA scores having a misclassification rate of IE in the low-risk group of < 4% and an NPV of >98%.

**Table 3:** Diagnostic accuracies of the fifth day PREDICT, VIRSTA, POSITIVE, and LAUSTAPHEN in predicting definite IE (according to the 2015 ESC-modified Duke Criteria).

**Scores**	**Patients**	**Sensitivity %** **(95% CI)**	**Specificity %** **(95% CI)**	**PPV %** **(95% CI)**	**NPV %** **(95% CI)**	**Accuracy %** **(95% CI)**	**PLR**	**NLR**	**TOE needed %**	**Endocarditis misclassified as low risk**
PREDICT (day 5) (all patients)	821	86.4 (78.9–92.1)	51.4 (47.6–55.1)	23.0 (21.2–24.9)	95.8 (93.4–97.3)	56.4 (52.9–59.8)	1.78 (1.60–1.97)	0.26 (0.17–0.42)	54.1%	16 (13.6%)
VIRSTA (all patients)	821	96.6 (91.2–99.1)	38.1 (34.5–41.8)	20.8 (19.7–21.9)	98.5 (96.2–99.4)	46.5 (43.1–50.0)	1.56 (1.46–1.67)	0.09 (0.03–0.23)	66.9%	4 (3.4%)
POSITIVE (all patients)	783	76.5 (67.0–84.3)	74.7 (71.3–78.0)	31.2 (27.7–34.9)	95.5 (93.7–96.8)	75.0 (71.8–78.0)	3.03 (2.56–3.58)	0.31 (0.22–0.45)	31.9%	24 (23.5%)
LAUSTAPHEN (derivation cohort)	419	96.5 (87.9–99.6)	57.5 (52.2–62.6)	26.3 (23.9–28.9)	99.1 (96.4–99.8)	62.8 (57.9–67.4)	2.27 (1.99–2.58)	0.06 (0.02–0.24)	49.9%	2 (3.5%)
LAUSTAPHEN (validation cohort)	402	96.7 (88.7–99.6)	54.3 (48.8–59.6)	27.4 (25.0–30.0)	98.9 (95.9–99.7)	60.7 (55.7–65.5)	2.11 (1.87–2.39)	0.06 (0.02–0.24)	53.5%	2 (3.3%)

ESC, European Society of Cardiology; IE, infective endocarditis; NLR, negative likelihood ratio; NPV, negative predictive value; PLR, positive likelihood ratio; PPV, positive predictive value; TOE, transoesophageal echocardiography.

**Table 4:** Diagnostic accuracies of the fifth day PREDICT, VIRSTA, POSITIVE, and LAUSTAPHEN in predicting cardiac lesions (according to imaging and pathological 2015 ESC-modified Duke Criteria).

**Scores**	**Patients**	**Sensitivity %** **(95% CI)**	**Specificity %** **(95% CI)**	**PPV %** **(95% CI)**	**NPV %** ** (95% CI)**	**Accuracy %** **(95% CI)**	**PLR**	**NLR**	**TOE needed %**	**Endocarditis misclassified as low risk**
PREDICT (day 5) (all patients)	821	87.3 (79.2–93.0)	50.5 (46.9–54.3)	20.1 (18.4–21.8)	96.6 (94.4–97.9)	55.2 (51.7–58.6)	1.77 (1.59–1.96)	0.25 (0.15–0.42)	54.1%	13 (13.6%)
VIRSTA (all patients)	821	96.6 (91.2–99.1)	38.1 (34.5–41.8)	20.8 (19.7–21.9)	98.5 (96.2–99.4)	46.5 (43.1–50.0)	1.53 (1.43–1.64)	0.11 (0.04–0.28)	66.9%	4 (3.4%)
POSITIVE (all patients)	783	71.3 (60.6–80.5)	73.0 (69.5–76.3)	24.8 (21.6–28.3)	95.3 (93.6–96.6)	72.8 (69.5–75.9)	2.64 (2.20–3.16)	0.39 (0.28–0.55)	31.9%	25 (28.7%)
LAUSTAPHEN (derivation cohort)	419	96.1 (86.5–99.5)	56.5 (51.3–61.7)	23.4 (21.2–25.8)	99.1 (96.4–99.8)	61.3 (56.5–66.0)	2.21 (1.94–2.51)	0.07 (0.02–0.27)	49.9%	2 (3.9%)
LAUSTAPHEN (validation cohort)	402	96.1 (86.5–99.5)	52.7 (47.4–58.0)	20.7 (20.7–25.0)	98.9 (96.0–99.7)	58.2 (53.2–63.1)	2.03 (1.80–2.30)	0.07 (0.02–0.29)	53.5%	2 (3.9%)

ESC, European Society of Cardiology; NLR, negative likelihood ratio; NPV, negative predictive value; PLR, positive likelihood ratio; PPV, positive predictive value; TOE, transoesophageal echocardiography.

**Figure 1:**
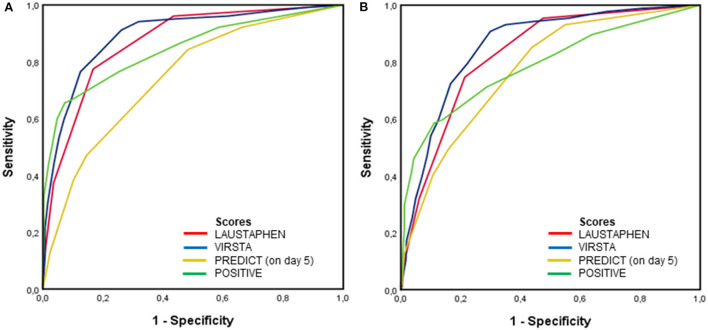
ROC of PREDICT, VIRSTA, POSITIVE, and LAUSTAPHEN scores in predicting **(A)** definite IE in the whole population and **(B)** cardiac lesion in patients who had an echocardiogram, cardiac surgery, or autopsy.

Figure 1 shows the ROC of scores in predicting definite IE in the whole study population (1A) and cardiac lesions in patients who had an echocardiogram, cardiac surgery, or autopsy (1B). The area under the curve (AUC) for predicting definite IE by LAUSTAPHEN, VIRSTA, PREDICT, and POSITIVE was 0.87, 0.89, 0.74, and 0.84, respectively. AUC for predicting cardiac lesion by LAUSTAPHEN, VIRSTA, PREDICT, and POSITIVE was 0.83, 0.86, 0.77, and 0.79, respectively.

The diagnostic accuracies of the aforementioned scores were additionally tested among the subgroup of patients that had all appropriate imaging investigations according to their Group allocation (patients with TOE in Groups 1 and 2, patients with TTE or TOE in Group 3, all patients in Group 4; Supplementary Table 2) are depicted in Supplementary Tables 5, 6. Supplementary Table 7 shows the diagnostic accuracies of the aforementioned scores in patients belonging to Groups 1 and 2 (high risk). In high-risk patients, LAUSTAPHEN and VIRSTA scores had a misclassification rate of IE in the low-risk group of < 1% and an NPV of >98%, but VIRSTA would have required more cardiac imaging investigations (TOE in 81.0% episodes) compared to LAUSTAPHEN (TOE in 64.9%) to achieve the same result. Supplementary Table 8 shows the diagnostic accuracies of the aforementioned scores in patients that had at least one cardiac imaging study (TTE, TOE, PET-CT, or cardiac CT) performed. In high-risk patients, LAUSTAPHEN and VIRSTA scores had a misclassification rate of IE in the low-risk group of 3.4% and an NPV of ≥98%, but VIRSTA would have required more cardiac imaging investigations (TOE in 72.0% episodes) compared to LAUSTAPHEN (TOE in 58.0%) to achieve the same result.

## Discussion

In the present study, the rate of definite IE among SAB (14.4%) was similar to that reported in previous studies (6.7–18.2%) (7–11). We developed and validated LAUSTAPHEN, a new prediction score of IE among patients with SAB. The primary aim of these scores is to reliably identify patients with SAB at low risk for IE, in order to avoid further cardiac imaging studies. Among the evaluated cores, only LAUSTAPHEN and VIRSTA achieved the specified threshold (< 4%) of misclassified episodes with IE in the low-risk group.

van der Vaart et al. (7) used an NPV above 98% as a threshold to consider a score safe for the exclusion of IE. In this study, beyond high NPV, we included a low false-negative rate (< 4%) for the evaluation of the scores. LAUSTAPHEN and VIRSTA scores achieved such criteria and both scores had an NLR of < 0.1, underlying their importance as good “rule-out” scores. These results are in line with previous studies showing that VIRSTA has an NPV above 98% (7–9); although, in a recent study, VIRSTA failed by a little to achieve that threshold (NPV of 97.8%) (20). Day 5 PREDICT score had an NPV of 96% which is comparable to previous studies, including the study that proposed a score of 94.5–97.9% (7, 9, 19, 20). However, our results are in contrast to the study that validated the PREDICT score which showed an NPV of 100% (11). Such difference might be explained by the low number of patients included in that study (*n* = 199), when compared to our cohort (*n* = 821) (11). Importantly, the high misclassification rate of IE episodes in the low-risk group of PREDICT (13.6%) and POSITIVE (23.5%) in this study rendered them inapplicable in clinical practice (7, 9, 10, 20).

Predictors included in PREDICT, POSITIVE, and VIRSTA scores were identified by multivariable regression models (19, 24). Although statistically robust, this approach might result in several inconsistencies. As an example, PREDICT does not include the presence of prosthetic valves or vascular phenomena as predictors (19). A previous meta-analysis recognized the presence of embolic events as the most important factor in patients with SAB to predict IE (15). Accordingly, a patient with SAB and an embolic event should be considered as having IE until proven otherwise and TOE is indicated, even if PREDICT score is < 2. Furthermore, the POSITIVE score does not include prolonged bacteremia as a predictor; the absence of that predictor might be explained by the excellent performance of the time to blood culture positivity in that study; all patients with IE in the POSITIVE cohort had a time to positivity inferior to 15 h (10). In this study, only 81.4% reached that threshold. Moreover, the POSITIVE score cannot be applied to all SAB since the calculation of the time to positivity might not be available, for example in patients with bacteremia onset in other hospitals. Time to positivity might also be unreliable in the context of polymicrobial bacteremia or in patients under antimicrobial treatment when initial blood cultures are collected (10). Finally, one of the 10 criteria included in the VIRSTA score is the presence of meningitis, which was also identified by their multivariable regression model. Nevertheless, meningitis accounted for < 2% of SAB, making its impact on clinical decisions minimal (8).

Even though the addition of native bone and joint infections as a fifth item of the LAUSTAPHEN score might seem arbitrary, it was based on the established association between native bone and joint infections with IE. IE prevalence among patients with bacteraemic native bone and joint infections due to *S. aureus* could reach 33% (25–27); thus, the presence of vertebral and non-vertebral osteomyelitis is part of the previous criteria for classifying patients at high risk for IE (14) or VIRSTA score (7).

Another important aspect of this study was that predictive scores were evaluated not only on their diagnostic accuracy but also on their clinical implications. In previous studies, TOE was performed in 30–50% of the cases, a rate similar to the present study (42.1% in the whole population and 50.7% in the high-risk group according to internal guidelines) (7–10, 20). If the scores were to be implemented in clinical practice, the number of TOEs needed to safely exclude IE would have increased to 66.9% with VIRSTA; this result is consistent with van der Vaart et al. (7) who showed that VIRSTA tended to overestimate the risk of IE. On the contrary, LAUSTAPHEN categorized only 51.6% of the included population into the high-risk group, reducing the number of TOEs needed as compared to VIRSTA.

Another advantage of LAUSTAPHEN is the limited number of variables to assess; only five items known to be associated with IE need to be evaluated, while VIRSTA score is based on 10 different parameters (8). The absence of complex calculations (presence of any criterion of the LAUSTAPHEN score categorizes the patient into the high-risk group) which are necessary for other scores (VIRSTA, POSITIVE, and PREDICT), renders LAUSTAPHEN an easy and practical score (8, 10, 19).

This study has several limitations. First, it is monocentric and retrospective, even though the number of included patients exceeded that of many previous studies (7, 10, 11). Second, despite internal guidelines, 14.7% of episodes did not undergo any cardiac imaging study (TTE, TOE, PET-CT, or cardiac CT), with 9.1% belonging to the high-risk group and 30.7% belonging to the low-risk group. However, in the 90 days following the initial SAB, only a small proportion of patients (2.1%) had a SAB relapse and only one patient developed *S. aureus* IE. Considering the aforementioned elements, we decided to include all patients, even those who did not undergo echocardiography and therefore were less likely to have IE. We performed subgroup analyses including only patients who had all imaging investigations considered appropriate according to their group allocation (Supplementary Tables 5, 6) and in patients that had at least one cardiac imaging study independently of their group allocation (Supplementary Table 8), where LAUSTAPHEN confirmed a false-negative rate inferior to 4%. Third, even though a cardiac imaging study was not performed for some patients, others had a second TTE and/or TOE performed due to high clinical suspicion; although this attitude was in accordance with 2015 ESC guidelines, it added to the selection bias created by the internal policy on the management of SAB (12). Fourth, LAUSTAPHEN and other scores included multiple variables (embolic events, cardiac predisposing conditions) that are also part of the reference standard (minor criteria for definite IE according to the 2015 ESC-modified Duke Criteria), possibly increasing the diagnostic accuracy for these predictors (8, 10, 19). To overcome this incorporation bias, we conducted a supplementary analysis using as a reference standard the presence of cardiac lesions representative of IE, which is less dependable from the variables included in the LAUSTAPHEN score. This analysis confirmed the low rate (< 4%) of misclassification of IE in the low-risk group and the high NPV (>98%) of the LAUSTAPHEN score. Fifth, although the LAUSTAPHEN score was validated in a different cohort than the derivation cohort, both originated from Lausanne University Hospital, which has an internal policy for SAB management that proposes that cardiac imaging studies are needed according to a pre-assessment of the IE risk based on the type of SAB (community vs. nosocomial) and common IE risk factors. The internal policy reflected common practice in many institutions as not all patients with SAB are at the same risk of acquiring IE (14, 15, 17). Therefore, it should be tested on an external patient population before being considered for application in clinical practice. Finally, in our hospital, all patients with SAB were examined by an infectious diseases specialist on the day of blood culture positivity, which led to improved clinical detection of embolic lesions not previously described by the treating physician and to a more systematic prescription of additional imaging studies for embolic foci detection. This could explain the high percentage of embolic events found in the present study (16.2%) as compared to previous studies (4.5–6.0%) (8, 9). Thus, the results of the present study cannot be extrapolated to centers in which infectious disease consultation is not mandatory for SAB.

In conclusion, we developed and validated a new reliable and easy-to-assess score including only five variables known to be associated with IE. Compared to POSITIVE and PREDICT scores, LAUSTAPHEN seemed more appropriate for clinical practice, due to its much lower misclassification rate of IE episodes in the low-risk group. Although LAUSTAPHEN and VIRSTA scores exhibited the lowest misclassification rate, VIRSTA significantly overestimated IE risk, leading to a higher number of echocardiograms (an increase of 29.7%) needed to achieve the same result as LAUSTAPHEN. Finally, no score is flawless; they might be used in conjunction with clinical judgment to help physicians to better guide further investigations.

## Data availability statement

The raw data supporting the conclusions of this article will be made available by the authors, without undue reservation.

## Ethics statement

The studies involving human participants were reviewed and approved by Ethics Committee of the Canton of Vaud (CER-VD). Written informed consent for participation was not required for this study in accordance with the national legislation and the institutional requirements.

## Author contributions

BG and LS conceived the idea. MP-O and PM collected the patients' data, performed the analysis, and interpreted the results. BG supervised the project. MP-O wrote the manuscript. All authors contributed to manuscript revision, read, and approved the submitted version.
