# Erratum: The LAUsanne STAPHylococcus aureus ENdocarditis (LAUSTAPHEN) score: A prediction score to estimate initial risk for infective endocarditis in patients with S. aureus bacteremia

**DOI:** 10.3389/fcvm.2023.1171903

**Published:** 2023-03-06

**Authors:** Frontiers Production Office

**Affiliations:** Frontiers Media SA, Lausanne, Switzerland

**Keywords:** Staphylococcus aureus bacteraemia, infective endocarditis, transoesophageal echocardiography (TOE), bloodstream infection, risk stratification

An Erratum on The LAUsanne STAPHylococcus aureus ENdocarditis (LAUSTAPHEN) score: A prediction score to estimate initial risk for infective endocarditis in patients with S. aureus bacteremia By Papadimitriou-Olivgeris M, Monney P, Mueller L, Senn L and Guery B. (2022) Front. Cardiovasc. Med. 9:961579. doi: 10.3389/fcvm.2022.961579

An omission to the funding section of the original article was made in error. The following sentence has been added: “Open access funding was provided by the University of Lausanne”.

The original version of this article has been updated.

