# Supplementary material for: The LAUsanne STAPHylococcus aureus ENdocarditis (LAUSTAPHEN) score: A prediction score to estimate initial risk for infective endocarditis in patients with S. aureus bacteremia
Source: Front Cardiovasc Med. 2022 Dec 9;9:961579. doi: 10.3389/fcvm.2022.961579 (PMC9780492; doi:10.3389/fcvm.2022.961579)
Supplement: Supplementary file 1 [file Data_Sheet_1.docx]

Supplementary Material


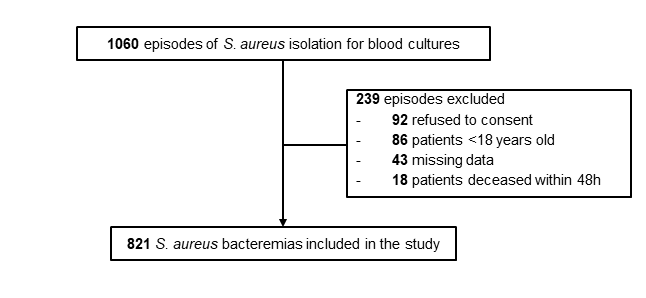


**Supplementary Figure 1.** Flowchart

**Supplementary Table 1.** Baseline characteristics of patients with *S. aureus* bacteraemia in the derivation and validation cohorts

|  | **Derivation cohort (n=419)** | | **Validation cohort**  **(n=402)** | | ***P*** |
| --- | --- | --- | --- | --- | --- |
| Demographics |  |  |  |  |  |
| Male sex | 307 | 73.3% | 278 | 69.2% | 0.193 |
| Age (years) | 67 | 54-77 | 68 | 45-78 | 0.400 |
| Co-morbidities |  |  |  |  |  |
| Congestive heart failure | 37 | 8.8% | 20 | 5.0% | 0.030 |
| Chronic obstructive pulmonary disease | 49 | 11.7% | 45 | 11.2% | 0.822 |
| Cirrhosis | 36 | 8.6% | 37 | 9.2% | 0.758 |
| Diabetes mellitus | 118 | 28.2% | 126 | 31.3% | 0.319 |
| Chronic kidney disease (moderate or severe) | 95 | 22.7% | 98 | 24.4% | 0.565 |
| Malignancy (solid organ or hematologic) | 76 | 18.1% | 78 | 19.4% | 0.643 |
| Obesity | 100 | 23.9% | 111 | 27.6% | 0.220 |
| Immunosuppression | 76 | 8.1% | 70 | 17.4% | 0.786 |
| Charlson Comorbidity Index | 5 | 3-7 | 5 | 3-7 | 0.333 |
| Setting of infection onset |  |  |  |  |  |
| Community | 178 | 42.5% | 174 | 43.3% | 0.817^a^ |
| Non-nosocomial healthcare-associated | 96 | 22.9% | 77 | 19.2% |  |
| Nosocomial | 145 | 34.6% | 151 | 37.6% |  |
| Cardiac predisposing factors | 60 | 14.3% | 76 | 18.9% | 0.077 |
| IV drug use | 36 | 8.6% | 36 | 9.0% | 0.854 |
| Prior endocarditis | 5 | 1.7% | 12 | 3.0% | 0.250 |
| Native valve disease | 4 | 1.0% | 6 | 1.5% | 0.539 |
| Prosthetic valve | 21 | 5.0% | 30 | 7.5% | 0.146 |
| Cardiac implantable electronic devices | 47 | 11.2% | 41 | 10.2% | 0.637 |
| Pacemaker | 26 | 6.2% | 25 | 6.2% |  |
| Defibrillator | 11 | 2.6% | 15 | 3.7% |  |
| Other | 10 | 2.4% | 1 | 0.2% |  |
| Presence of prosthetic material (other than cardiac valve) |  |  |  |  |  |
| Endovascular (non-cardiac) prosthetic material | 26 | 6.2% | 21 | 5.2% | 0.545 |
| Bone or joint prosthetic material | 94 | 22.4% | 93 | 23.1% | 0.811 |
| Microbiological data |  |  |  |  |  |
| Two or more blood cultures positive | 332 | 79.2% | 310 | 77.1% | 0.462 |
| Polymicrobial bacteraemia | 40 | 9.5% | 32 | 8.0% | 0.422 |
| Methicillin-resistance | 34 | 8.1% | 30 | 7.5% | 0.728 |
| Time to blood culture positivity (h) (among 783 patients) | 13 | 10-17 | 13 | 10-17 | 0.761 |
| Time to blood culture positivity <9h | 72 | 18.2% | 74 | 19.1% | 0.740^b^ |
| Time to blood culture positivity 9-11h | 92 | 23.2% | 71 | 18.3% |  |
| Time to blood culture positivity 11-13 hours | 61 | 15.4% | 61 | 15.8% |  |
| Duration of bacteraemia (h) | 0 | 0-50 | 0 | 0-47 | 0.250 |
| Prolonged bacteraemia ≥48h | 108 | 25.8% | 99 | 24.6% | 0.705 |
| Prolonged bacteraemia ≥72h | 69 | 16.5% | 66 | 16.4% | 0.985 |
| Imaging criterion | 45 | 10.7% | 44 | 10.9% | 0.925 |
| TTE performed | 317 | 75.5% | 319 | 79.4% | 0.205 |
| TEO performed | 169 | 40.3% | 177 | 44.0% | 0.284 |
| 18-FDG PET-CT or cardiac CT performed | 58 | 13.8% | 46 | 11.4% | 0.301 |
| Any cardiac imaging performed | 352 | 84.0% | 348 | 86.6% | 0.301 |
| Groups (according to internal policy) |  |  |  |  |  |
| Group 1 (high-risk) | 274 | 65.4% | 251 | 62.4% | 0.377^c^ |
| Group 2 (high-risk) | 40 | 9.5% | 41 | 10.2% |  |
| Group 3 (low-risk) | 47 | 11.2% | 49 | 12.2% |  |
| Group 4 (low-risk) | 58 | 13.8% | 61 | 15.2% |  |
| Infection data |  |  |  |  |  |
| Duration of systemic symptoms (days) | 1 | 1-3 | 1 | 1-2 | 0.047 |
| Fever | 345 | 82.3% | 343 | 85.3% | 0.246 |
| Heart murmur | 137 | 32.7% | 119 | 29.6% | 0.339 |
| New heart murmur | 86 | 20.5% | 90 | 22.4% | 0.516 |
| Vascular phenomena | 68 | 16.2% | 64 | 15.9% | 0.904 |
| Location |  |  |  |  |  |
| Limbs | 15 | 3.6% | 15 | 3.7% | 0.908 |
| Trunk | 49 | 11.7% | 42 | 10.4% | 0.569 |
| Cerebral | 26 | 6.2% | 38 | 9.5% | 0.083 |
| Type |  |  |  |  |  |
| Ischemic stroke | 22 | 5.3% | 32 | 8.0% | 0.117 |
| Hemorrhagic stroke | 4 | 1.0% | 8 | 2.0% | 0.255 |
| Cerebral mycotic aneurysm | 0 | 0.0% | 4 | 1.0% | 0.057 |
| Janeway lesions | 9 | 2.1% | 10 | 2.5% | 0.746 |
| Nail bed hemorrhage | 2 | 0.5% | 2 | 0.5% | 1.000 |
| Conjunctival bleeding | 0 | 0.0% | 0 | 0.0% | - |
| Septic lung emboli | 25 | 6.0% | 23 | 5.7% | 0.881 |
| Renal emboli | 14 | 3.3% | 12 | 3.0% | 0.771 |
| Splenic emboli | 17 | 4.1% | 16 | 4.0% | 0.955 |
| Non-cerebral mycotic aneurysm | 12 | 2.9% | 7 | 1.7% | 0.285 |
| Arterial emboli | 3 | 0.7% | 2 | 0.5% | 1.000 |
| Other foci of infection |  |  |  |  |  |
| Meningitis | 2 | 0.5% | 5 | 1.2% | 0.278 |
| Bone and joint infection (excluding chronic osteomyelitis) | 107 | 25.5% | 107 | 26.6% | 0.725 |
| Native bone and joint infection (excluding chronic osteomyelitis) | 81 | 19.3% | 74 | 18.4% | 0.735 |
| Native septic arthritis | 44 | 10.5% | 45 | 11.2% | 0.750 |
| Vertebral osteomyelitis | 39 | 9.3% | 27 | 6.7% | 0.172 |
| Acute non-vertebral osteomyelitis | 8 | 1.9% | 6 | 1.5% | 0.645 |
| Prosthetic bone and joint infection | 31 | 7.4% | 38 | 9.5% | 0.289 |
| Prosthetic joint infection | 25 | 6.0% | 28 | 7.0% | 0.561 |
| Osteosynthesis or spondylodesis infection | 6 | 1.4% | 10 | 2.5% | 0.274 |
| Immunologic phenomena | 7 | 1.7% | 8 | 2.0% | 0.733 |
| Sepsis | 177 | 42.2% | 160 | 39.8% | 0.49 |
| Septic shock | 62 | 14.8% | 64 | 15.9% | 0.655 |
| Laboratory data |  |  |  |  |  |
| White blood cells (×10^9^/l) | 12.4 | 8.2-16.9 | 12.3 | 9.4-16.0 | 0.841 |
| CRP (mg/l) (among 762 patients) | 199 | 103-296 | 203 | 101-310 | 0.469 |
| CRP≥190mg/l | 207 | 52.8% | 200 | 54.1% | 0.703 |
| Management |  |  |  |  |  |
| Infectious diseases consultation | 341 | 94.2% | 56 | 98.2% | 0.203 |
| Cardiac surgery | 20 | 4.8% | 16 | 4.0% | 0.579 |
| CIED removal (among 88 patients with CIED) | 13 | 27.7% | 16 | 39.0% | 0.258 |
| Autopsy (within 30 days) | 8 | 1.9% | 6 | 1.5% | 0.645 |
| Proven infective endocarditis based on clinical and pathological 2015 ESC modified Duke criteria | 57 | 13.6% | 61 | 15.2% | 0.521 |
| Cardiac lesion based on imaging and pathological 2015 ESC modified Duke criteria | 51 | 12.2% | 51 | 12.7% | 0.823 |

Data are depicted as number and percentage or median and Q1-3

^a^Comparison of community-acquired against both non-nosocomial healthcare-associated and nosocomial

^b^Comparison of time to blood culture positivity <9h against ≥9

^c^Comparison of Group 1 against all other Groups

18-FDG PET-CT: 18-fluorodeoxyglucose positron emission positron emission tomography computed tomography; CIED: cardiac implantable electronic devices; CRP: C-reactive protein; ESC: European Society of Cardiology; TTE: transthoracic echocardiography; TEO: transoesophageal echocardiography

**Supplementary Table 2.** Group repartition of patients with *S. aureus* bacteraemia depending on internal policy

|  | *S. aureus* bacteraemia (n=821) | | | |
| --- | --- | --- | --- | --- |
|  | High risk | | Low risk | |
|  | Group 1 (n=525) | Group 2 (n=81) | Group 3 (n=96) | Group 4 (n=119) |
| TTE performed | 437 (83.2%) | 66 (81.5%) | 57 (59.4%) | 76 (63.9%) |
| TEO performed | 273 (52.0%)^a^ | 34 (42.0%)^a^ | 23 (24.0%) | 16 (13.4%) |
| 18-FDG PET-CT or cardiac CT performed | 72 (13.7%) | 23 (28.4%) | 2 (2.1%) | 7 (5.9%) |
| Any imaging study performed | 475 (90.5%) | 76 (93.8%) | 67 (69.8%) | 82 (68.9%) |
| Definite endocarditis based on clinical and pathological 2015 ESC modified Duke criteria | 101 (19.2%) | 14 (17.3%) | 3 (3.1%) | 0 (0%) |
| Cardiac lesion based on imaging and pathological 2015 ESC modified Duke criteria | 86 (16.4%) | 13 (16.0%) | 3 (3.1%) | 0 (0%) |
| Bloodstream infection recurrence (same pathogen) within 90 days | 9 (1.7%) | 3 (3.7%) | 3 (3.1%) | 2 (1.7%) |
| Infectious endocarditis (same pathogen) within 90 days | 1 (0.2%) | 0 (0%) | 0 (0%) | 0 (0%) |

Group 1: community acquired (TTE and TEO indicated)

Group 2: nosocomial with risk factors (prior endocarditis, presence of CIED or prosthetic valve, persistent BSI for 72h, embolic event) (TTE and TEO indicated)

Group 3: nosocomial not catheter-related without risk factors (TTE indicated)

Group 4: nosocomial catheter-related without risk factors (no further investigation)

18-FDG PET-CT: 18-fluorodeoxyglucose positron emission positron emission tomography computed tomography; ESC: European Society of Cardiology; TTE: transthoracic echocardiography; TEO: transoesophageal echocardiography

^a^TOE was not performed in 299 episodes in Groups 1 and 2 because of: combination of age and comorbidities (100 episodes; 33.4%), contraindication (esophageal varices, severe thrombocytopenia) or non-feasibility (severe obesity, inability to pass the endoscope, death before TOE) (44; 14.7%), positivity of TTE (10; 3.3%); for the remaining 145 episodes (48.5%), the risk was deemed low by the treating physician or infectious diseases consultant, thus no further testing was pursued.

**Supplementary Table 3.** Distribution of 2015 ESC modified Duke criteria

| Modified Duke criteria | Number of patients | Number of patients with pathological criterion | Number of patients with imaging criterion | Number of patients with at least one cardiac imaging study |
| --- | --- | --- | --- | --- |
| 2 major | 86 | 37 | 86 | 86 (100%) |
| 1 major and ≥ 3minor | 21 | 3 | 1 | 19 (90.5%) |
| 1 major and 2 minor | 94 | 3 | 2 | 92 (79.9%) |
| 1 major and 1 minor | 371 | 6 | 0 | 314 (84.6%) |
| 1 major | 73 | 1 | 0 | 64 (87.7%) |
| 4 minor | 3 | 1 | 0 | 3 (100%) |
| 3 minor | 21 | 0 | 0 | 18 (85.7%) |
| 2 minor | 119 | 0 | 0 | 78 (65.5%) |
| 1 minor | 33 | 0 | 0 | 26 (78.8%) |

**Supplementary Table 4.** Predictors of cardiac lesion (according to imaging and pathological 2015 ESC modified Duke criteria) in patients with *S. aureus* bacteraemia in the derivation cohort

|  | **Univariate analysis** | | | | | **Multivariable analysis** | |
| --- | --- | --- | --- | --- | --- | --- | --- |
|  | **Without IE (n=368)** | | **IE (n=51)** | | ***P*** | ***P*** | **OR (95% CI)** |
| Demographics |  |  |  |  |  |  |  |
| Male sex | 267 | 72.6% | 40 | 78.4% | 0.374 |  |  |
| Age (years) | 67 | 55-79 | 61 | 49-71 | 0.012 |  |  |
| Co-morbidities |  |  |  |  |  |  |  |
| Congestive heart failure | 30 | 8.2% | 7 | 13.7% | 0.189 |  |  |
| Chronic obstructive pulmonary disease | 41 | 11.1% | 8 | 15.7% | 0.344 |  |  |
| Cirrhosis | 34 | 9.2% | 2 | 3.9% | 0.288 |  |  |
| Diabetes mellitus | 109 | 29.6% | 9 | 17.6% | 0.075 |  |  |
| Chronic kidney disease (moderate or severe) | 84 | 22.8% | 11 | 21.6% | 0.841 |  |  |
| Malignancy (solid organ or hematologic) | 74 | 20.1% | 2 | 3.9% | 0.003 |  |  |
| Obesity | 85 | 23.1% | 15 | 29.4% | 0.2322 |  |  |
| Immunosuppression | 75 | 20.4% | 1 | 2.0% | <0.001 |  |  |
| Charlson Comorbidity Index | 5 | 3-7 | 3 | 2-7 | 0.008 |  |  |
| Setting of infection onset |  |  |  |  |  |  |  |
| Community | 145 | 39.4% | 33 | 64.7% | 0.001^a^ |  |  |
| Non-nosocomial healthcare-associated | 87 | 23.6% | 9 | 17.6% |  |  |  |
| Nosocomial | 136 | 37.0% | 9 | 17.6% |  |  |  |
| Cardiac predisposing factors^a^ | 37 | 10.1% | 30 | 58.8% | <0.001 | 0.002 | 3.5 (1.6-7.7) |
| IV drug use | 28 | 7.6% | 8 | 15.7% | 0.054 |  |  |
| Prior endocarditis | 5 | 1.4% | 2 | 3.9% | 0.205 |  |  |
| Native valve disease | 2 | 0.5% | 2 | 3.9% | 0.074 |  |  |
| Prosthetic valve | 9 | 2.4% | 12 | 23.5% | <0.001 |  |  |
| Cardiac implantable electronic devices | 30 | 8.2% | 17 | 33.3% | <0.001 | <0.001 | 5.8 (2.5-13.7) |
| Pacemaker | 17 | 4.6% | 9 | 17.6% |  |  |  |
| Defibrillator | 6 | 1.6% | 5 | 9.8% |  |  |  |
| Other | 7 | 1.9% | 3 | 5.9% |  |  |  |
| Presence of prosthetic material (other than cardiac valve) |  |  |  |  |  |  |  |
| Endovascular (non-cardiac) prosthetic material | 19 | 5.2% | 7 | 13.7% | 0.027 |  |  |
| Bone or joint prosthetic material | 84 | 22.8% | 10 | 19.6% | 0.606 |  |  |
| Microbiological data |  |  |  |  |  |  |  |
| Two or more blood cultures positive | 282 | 76.6% | 50 | 98.0% | <0.001 |  |  |
| Polymicrobial bacteraemia | 37 | 10.1% | 3 | 5.9% | 0.451 |  |  |
| Methicillin-resistance | 33 | 9.0% | 1 | 2.0% | 0.102 |  |  |
| Time to blood culture positivity (h) (among 396 patients) | 13 | 10-17 | 11 | 8-15 | 0.017 |  |  |
| Time to blood culture positivity <9h | 54 | 15.2% | 18 | 43.9% | <0.001^b^ |  |  |
| Time to blood culture positivity 9-11h | 81 | 22.8% | 11 | 26.8% |  |  |  |
| Time to blood culture positivity 11-13 hours | 57 | 16.1% | 4 | 9.8% |  |  |  |
| Duration of bacteraemia (h) | 0 | 0-41 | 65 | 28-110 | <0.001 |  |  |
| Prolonged bacteraemia ≥48h | 79 | 21.5% | 29 | 56.9% | <0.001 | 0.029 | 1.3 (1.0-1.7) |
| Prolonged bacteraemia ≥72h | 48 | 13.0% | 21 | 41.2% | <0.001 |  |  |
| Imaging criterion | 0 | 0.0% | 89 | 87.3% | <0.001 |  |  |
| TTE performed | 276 | 75.0% | 41 | 80.4% |  |  |  |
| TOE performed | 128 | 34.8% | 41 | 80.4% |  |  |  |
| 18-FDG PET-CT or cardiac CT performed | 43 | 11.7% | 15 | 29.4% |  |  |  |
| Any cardiac imaging performed | 302 | 82.1% | 50 | 98.0% |  |  |  |
| Infection data |  |  |  |  |  |  |  |
| Duration of systemic symptoms (days) | 1 | 1-2 | 2 | 1-3 | <0.001 |  |  |
| Fever | 301 | 81.8% | 44 | 86.3% | 0.432 |  |  |
| Heart murmur | 107 | 29.1% | 30 | 58.8% | <0.001 |  |  |
| New heart murmur | 63 | 17.1% | 23 | 45.1% | <0.001 |  |  |
| Vascular phenomena | 36 | 9.8% | 32 | 62.7% | <0.001 | <0.001 | 8.9 (4.2-18.7) |
| Location |  |  |  |  |  |  |  |
| Limbs | 6 | 1.6% | 9 | 17.6% | <0.001 |  |  |
| Trunk | 27 | 7.3% | 22 | 43.1% | <0.001 |  |  |
| Cerebral | 8 | 2.2% | 18 | 35.3% | <0.001 |  |  |
| Type |  |  |  |  |  |  |  |
| Ischemic stroke | 7 | 1.9% | 15 | 29.4% | <0.001 |  |  |
| Hemorrhagic stroke | 1 | 0.3% | 3 | 5.9% | 0.006 |  |  |
| Cerebral mycotic aneurysm | 0 | 0.0% | 0 | 0.0% | - |  |  |
| Janeway lesions | 2 | 0.5% | 7 | 13.7% | <0.001 |  |  |
| Nail bed hemorrhage | 0 | 0.0% | 2 | 3.9% | 0.015 |  |  |
| Conjunctival bleeding | 0 | 0.0% | 0 | 0.0% | - |  |  |
| Septic lung emboli | 15 | 4.1% | 10 | 19.6% | <0.001 |  |  |
| Renal emboli | 5 | 1.4% | 9 | 17.6% | <0.001 |  |  |
| Splenic emboli | 6 | 1.6% | 11 | 21.6% | <0.001 |  |  |
| Non-cerebral mycotic aneurysm | 11 | 3.0% | 1 | 2.0% | 1.000 |  |  |
| Arterial emboli | 1 | 0.3% | 2 | 3.9% | 0.040 |  |  |
| Other foci of infection |  |  |  |  |  |  |  |
| Meningitis | 0 | 0.0% | 2 | 3.9% | 0.015 |  |  |
| Bone and joint infection (excluding chronic osteomyelitis) | 89 | 24.2% | 18 | 35.3% | 0.088 |  |  |
| Native bone and joint infection (excluding chronic osteomyelitis) | 63 | 17.1% | 18 | 35.3% | 0.002 | 0.125 | 1.9 (0.8-4.4) |
| Native septic arthritis | 33 | 9.0% | 11 | 21.6% | 0.012 |  |  |
| Vertebral osteomyelitis | 31 | 8.4% | 8 | 15.7% | 0.094 |  |  |
| Acute non-vertebral osteomyelitis | 6 | 1.6% | 2 | 3.9% | 0.253 |  |  |
| Prosthetic bone and joint infection | 30 | 8.2% | 1 | 2.0% | 0.154 |  |  |
| Prosthetic joint infection | 25 | 6.8% | 0 | 0.0% | 0.057 |  |  |
| Osteosynthesis or spondylodesis infection | 5 | 1.4% | 1 | 2.0% | 0.543 |  |  |
| Immunologic phenomena | 3 | 0.8% | 4 | 7.8% | 0.005 |  |  |
| Sepsis | 148 | 40.2% | 29 | 56.9% | 0.024 |  |  |
| Septic shock | 43 | 11.7% | 19 | 37.3% | <0.001 |  |  |
| Laboratory data |  |  |  |  |  |  |  |
| White blood cells (×10^9^/l) | 12.2 | 7.9-17.0 | 12.8 | 10.5-16.0 | 0.280 |  |  |
| CRP (mg/l) (among 392 patients) | 194 | 95-289 | 250 | 154-320 | 0.004 |  |  |
| CRP≥190mg/l | 175 | 50.9% | 32 | 66.7% | 0.040 |  |  |
| Management |  |  |  |  |  |  |  |
| Infectious diseases consultation | 347 | 94.3% | 40 | 98.0% | 0.261 |  |  |
| Cardiac surgery | 0 | 0.0% | 20 | 39.2% | <0.001 |  |  |
| CIED removal (among 47 patients with CIED) | 4 | 13.3% | 9 | 52.9% | 0.006 |  |  |
| Autopsy (within 30 days) | 5 | 1.4% | 3 | 5.9% | 0.061 |  |  |

Data are depicted as number and percentage or median and Q1-3

^a^Comparison of community-acquired against both non-nosocomial healthcare-associated and nosocomial

^b^Comparison of time to blood culture positivity <9h against ≥9

18-FDG PET-CT: 18-fluorodeoxyglucose positron emission positron emission tomography computed tomography; CIED: cardiac implantable electronic devices; CRP: C-reactive protein; ESC: European Society of Cardiology; IE: infective endocarditis; TTE: transthoracic echocardiography; TOE: transoesophageal echocardiography

**Supplementary Table 5.** Diagnostic accuracies of 5^th^ day PREDICT, VIRSTA, POSITIVE and LAUSTAPHEN in predicting definite endocarditis (according to 2015 ESC modified Duke criteria) among all patients that had appropriate imaging studies depending on their Group

| **Scores** | **Patients** | **Sensitivity**  **% (95% CI)** | **Specificity**  **% (95% CI)** | **PPV**  **% (95% CI)** | **NPV**  **% (95% CI)** | **Accuracy**  **% (95% CI)** | **PLR** | **NLR** | **TEO needed**  **%** | **Endocarditis misclassified as low risk** |
| --- | --- | --- | --- | --- | --- | --- | --- | --- | --- | --- |
| **PREDICT (day 5)** | 540 | 86.6 (78.9-92.3) | 56.1 (51.2-60.8) | 34.0 (31.2-37.0) | 94.1 (90.8-96.3) | 62.4 (58.2-66.5) | 1.97 (1.73-2.24) | 0.24 (0.15-0.39) | 52.8% | 15 (13.4%) |
| **VIRSTA** | 540 | 96.4 (91.1-99.0) | 44.2 (39.4-49.0) | 31.1 (29.2-33.1) | 97.9 (94.7-99.2) | 55.0 (50.7-59.3) | 1.73 (1.58-1.89) | 0.08 (0.03-0.21) | 64.3% | 4 (3.6%) |
| **POSITIVE** | 508 | 75.0 (65.1-83.3) | 73.1 (68.5-77.3) | 39.3 (34.8-44.1) | 92.6 (89.8-94.7) | 73.4 (69.4-77.2) | 2.78 (2.29-3.39) | 0.34 (0.24-0.49) | 36.0% | 24 (25.0%) |
| **LAUSTAPHEN** | 540 | 96.4 (91.1-99.0) | 53.0 (48.2-99.0) | 35.0 (32.6-37.4) | 98.3 (95.6-99.3) | 62.0 (57.8-66.2) | 2.05 (1.85-2.28) | 0.07 (0.03-0.18) | 57.2% | 4 (3.6%) |

ESC: European Society of Cardiology; NLR: negative likelihood ratio; NPV: negative predictive value; PLR: positive likelihood ratio; PPV: positive predictive value; TEO: transoesophageal echocardiography

**Supplementary Table 6.** Diagnostic accuracies of 5th day PREDICT, VIRSTA, POSITIVE and LAUSTAPHEN in predicting cardiac lesion (according to imaging and pathological 2015 ESC modified Duke criteria) among all patients that had an appropriate imaging study depending on their Group

| **Scores** | **Patients** | **Sensitivity**  **% (95% CI)** | **Specificity**  **% (95% CI)** | **PPV**  **% (95% CI)** | **NPV**  **% (95% CI)** | **Accuracy**  **% (95% CI)** | **PLR** | **NLR** | **TEO needed**  **%** | **Endocarditis misclassified as low risk** |
| --- | --- | --- | --- | --- | --- | --- | --- | --- | --- | --- |
| **PREDICT (day 5)** | 540 | 87.3 (79.2-93.0) | 55.3 (50.5-60.0) | 31.2 (28.6-34.0) | 94.9 (91.8-96.9) | 61.3 (57.0-65.4) | 1.95 (1.72-2.22) | 0.23 (0.14-0.39) | 52.8% | 13 (12.7%) |
| **VIRSTA** | 540 | 96.1 (90.3-98.9) | 43.2 (38.5-47.9) | 28.2 (26.4-30.1) | 97.9 (94.7-99.2) | 53.2 (48.8-57.4) | 1.69 (1.54-1.85) | 0.09 (0.03-0.24) | 64.3% | 4 (3.9%) |
| **POSITIVE** | 508 | 71.3 (60.6-80.5) | 71.3 (66.7-75.5) | 33.9 (29.5-38.5) | 92.3 (89.6-94.4) | 71.3 (67.1-75.2) | 2.48 (2.03-3.03) | 0.40 (0.29-0.56) | 36.0% | 25 (28.7%) |
| **LAUSTAPHEN** | 540 | 96.1 (90.3-98.9) | 51.8 (47.0-56.6) | 31.7 (29.5-34.0) | 98.3 (95.6-99.3) | 60.2 (55.9-64.3) | 1.99 (1.80-2.21) | 0.08 (0.03-0.20) | 57.2% | 4 (3.9%) |

ESC: European Society of Cardiology; NLR: negative likelihood ratio; NPV: negative predictive value; PLR: positive likelihood ratio; PPV: positive predictive value; TEO: transoesophageal echocardiography

**Supplementary Table 7.** Diagnostic accuracies of 5^th^ day PREDICT, VIRSTA, POSITIVE and LAUSTAPHEN in predicting definite endocarditis (according to 2015 ESC modified Duke criteria) among patients belonging in Groups 1 and 2 (high-risk)

| **Scores** | **Patients** | **Sensitivity**  **% (95% CI)** | **Specificity**  **% (95% CI)** | **PPV**  **% (95% CI)** | **NPV**  **% (95% CI)** | **Accuracy**  **% (95% CI)** | **PLR** | **NLR** | **TEO needed**  **%** | **Endocarditis misclassified as low risk** |
| --- | --- | --- | --- | --- | --- | --- | --- | --- | --- | --- |
| **PREDICT (day 5)** | 606 | 88.7 (81.5-93.8) | 30.4 (26.3-34.6) | 23.0 (21.5-24.6) | 92.0 (87.1-95.1) | 41.4 (37.5-45.5) | 1.27 (1.17-1.39) | 0.37 (0.22-0.63) | 73.3% | 13 (11.3%) |
| **VIRSTA** | 606 | 99.1 (95.3-100) | 16.1 (13.0-19.6) | 21.7 (21.0-22.4) | 98.8 (91.7-99.8) | 31.9 (28.2-35.7) | 1.18 (1.12-1.23) | 0.05 (0.01-0.38) | 81.0% | 1 (0.9%) |
| **POSITIVE** | 571 | 78.8 (69.4-86.4) | 70.8 (66.4-74.8) | 36.1 (32.2-40.2) | 94.1 (91.6-95.9) | 72.2 (68.3-75.8) | 2.69 (2.27-3.21) | 0.30 (0.20-0.44) | 37.8% | 21 (26.9%) |
| **LAUSTAPHEN** | 606 | 99.1 (95.3-100) | 43.2 (38.8-47.7) | 29.0 (27.4-30.7) | 99.5 (96.8-99.9) | 53.8 (49.7-57.8) | 1.74 (1.61-1.89) | 0.02 (<0.01-0.14) | 64.9% | 1 (0.9%) |

ESC: European Society of Cardiology; NLR: negative likelihood ratio; NPV: negative predictive value; PLR: positive likelihood ratio; PPV: positive predictive value; TEO: transoesophageal echocardiography

**Supplementary Table 8.** Diagnostic accuracies of 5^th^ day PREDICT, VIRSTA, POSITIVE and LAUSTAPHEN in predicting definite endocarditis (according to 2015 ESC modified Duke criteria) among patients that had at least one cardiac imaging study

| **Scores** | **Patients** | **Sensitivity**  **% (95% CI)** | **Specificity**  **% (95% CI)** | **PPV**  **% (95% CI)** | **NPV**  **% (95% CI)** | **Accuracy**  **% (95% CI)** | **PLR** | **NLR** | **TEO needed**  **%** | **Endocarditis misclassified as low risk** |
| --- | --- | --- | --- | --- | --- | --- | --- | --- | --- | --- |
| **PREDICT (day 5)** | 700 | 86.1 (78.6-91.9) | 46.9 (42.8-51.1) | 24.4 (21.9-27.1) | 94.5 (91.5-96.5) | 53.4 (49.7-57.2) | 1.62 (1.46-1.80) | 0.29 (0.19-0.47) | 58.6% | 16 (13.8%) |
| **VIRSTA** | 700 | 96.6 (91.4-99.1) | 32.9 (29.1-36.9) | 16.6 (21.1-23.4) | 98.0 (94.8-99.2) | 43.4 (39.7-47.2) | 1.44 (1.35-1.54) | 0.10 (0.04-0.28) | 72.0% | 4 (3.4%) |
| **POSITIVE** | 662 | 76.0 (66.4-84.) | 72.6 (68.0-76.3) | 33.0 (29.3-37.0) | 94.4 (92.3-96.0) | 73.1 (69.6-76.5) | 2.77 (2.33-3.30) | 0.33 (0.23-0.47) | 34.7% | 24 (24.0%) |
| **LAUSTAPHEN** | 700 | 96.6 (91.4-99.1) | 49.7 (45.5-53.8) | 27.6 (25.9-29.4) | 98.6 (96.5-99.5) | 57.4 (53.7-61.1) | 1.92 (1.76-2.09) | 0.07 (0.03-0.18) | 58.0% | 4 (3.4%) |

ESC: European Society of Cardiology; NLR: negative likelihood ratio; NPV: negative predictive value; PLR: positive likelihood ratio; PPV: positive predictive value; TEO: transoesophageal echocardiography
